# Antibacterial and antibiofilm activities of protocatechualdehyde and its synergy with ampicillin against methicillin-resistant *Staphylococcus aureus*

**DOI:** 10.3389/fmicb.2024.1366400

**Published:** 2024-02-28

**Authors:** Ying Wang, Xiaojing Liu, Lin Song, Kun Chen, Chao Shi, Chuantao Peng, Qingli Yang, Zhaojie Li

**Affiliations:** ^1^School of Food Science and Engineering, Qingdao Agriculture University, Qingdao, China; ^2^Special Food Research Institute, Qingdao Agricultural University, Qingdao, China; ^3^Weihai Vocational College, Weihai, Shandong, China; ^4^Shandong Provincial Key Laboratory of Biochemical Engineering, College of Marine Science and Biological Engineering, Qingdao University of Science and Technology, Qingdao, Shandong, China; ^5^Wuqiong Food Co., Ltd., Raoping, Guangdong, China; ^6^College of Food Science and Nutritional Engineering, China Agriculture University, Beijing, China; ^7^College of Food Science and Engineering, Northwest A&F University, Yangling, Shaanxi, China

**Keywords:** protocatechualdehyde, MRSA, antibacterial activity, antibiofilm activity, antibiotic resistance

## Abstract

Protocatechualdehyde (PA) is a phenolic acid present in many plants and has many biological activities. Herein, the antagonistic effects and the action mechanism of PA against methicillin-resistant *Staphylococcus aureus* (MRSA) were studied. The results showed that PA had both significant antibacterial and anti-biofilm activities against MRSA. Additionally, PA had synergy with ampicillin against MRSA. It was elucidated that PA was prominent in destroying cell membranes, increasing cell membrane permeability and intracellular ROS production, thus leading to bacterial cell damage. Transcriptome analysis showed that PA disrupts many physiological pathways, including increasing cell membrane permeability, inhibiting biofilm formation, decreasing resistance to antimicrobial agents, and impairing DNA replication. Finally, the antimicrobial preservation test showed that PA could inhibit the growth of MRSA and prevent the corruption of beef. In summary, PA is an effective natural antibacterial substance and has a good application potential in food preservation, even in tackling antibiotic resistance problems.

## Highlights

• Protocatechualdehyde (PA) is an effective natural antibacterial agent against MRSA.
• PA exerts anti-biofilm activity on MRSA and increases membrane permeability.
• PA and ampicillin can synergistically inhibit MRSA proliferation.
• PA interferes with the expression of many genes.
• PA has good application potential in the preservation of cooked beef.

## 1 Introduction

*Staphylococcus aureus* (*S. aureus*) is an important foodborne pathogen and nosocomial infectious bacteria that causes clinical bloodstream infections, including skin and tissue infections, pneumonia, endocarditis, chronic osteomyelitis, and scald skin syndrome (Jenkins et al., 2008). However, during the treatment of *S. aureus* infections with the mass use of antibiotics, such as penicillin and methicillin, *S. aureus* quickly becomes resistant to these antibiotics. Methicillin-resistant *S. aureus* (MRSA) is the most common drug-resistant strain, with high morbidity and mortality rates; it was first reported in the United Kingdom in 1961 and induces infections by colonizing the skin, and oral mucosa of healthy organisms (Monaco et al., 2017). The US Centers for Disease Control and Prevention (CDC) outlined the top 18 drug-resistant bacteria, including MRSA. Moreover, MRSA gradually developed multidrug resistance to many other conventional antibiotics, such as macrolides, linezolid, aminoglycosides, ampicillin streptomycin, sulfonamides, vancomycin, chloramphenicol, and tetracycline, posing serious challenges in the treatment of MRSA infections (Kali, 2015). MRSA infections have traditionally been confined to the hospital environment and named hospital-acquired MRSA (HA-MRSA). With the dynamic development of the epidemiology of MRSA infections, the emergence of a new strain of MRSA that has more virulence than that of HA-MRSA, known as community-acquired MRSA (CA-MRSA) (Senok et al., 2020). It is predicted that the number of deaths owing to multidrug-resistant infections will increase by up to 10 million worldwide by 2050, which will cause higher mortality than cancer and lead to severe threats to human health (Posada-Perlaza et al., 2019).

However, the mechanisms underlying MRSA resistance are complex. Antibiotic resistance is mainly mediated through a 21–67 kb mobile genetic element (MGE) called the staphylococcal chromosome cassette *mec* (*SCCmec*), which carries the *mecA* resistance genes. These two genes encode two penicillin-binding proteins called PBP2a and PBP2c, respectively, which have an extremely low affinity for β-lactam antibiotics, resulting in antibiotic resistance (Ito et al., 2012; Gitman et al., 2021). Additionally, with complex 3D structures, the formation of bacterial biofilms enables bacteria to develop high resistance to many chemical and physical factors, particularly antibiotics, making their antibiotic resistance 10–1,000 times higher than that of planktonic bacteria (Borges et al., 2012). Furthermore, under the regulation of the agr and sar systems, MRSA can form biofilms, thereby improving its antibiotic resistance (Qin et al., 2014). Finally, the high frequency of overexpression of the multidrug resistance efflux pump in MRSA indicates that the efflux pump may contribute to the antibiotic resistance of MRSA (Kosmidis et al., 2012). These combinations lead to the complexity of MRSA antibiotic resistance and the difficulty in treating MRSA infections.

Although using antibiotics is still one of the most important ways to fight against MRSA infections, the excessive use of antibiotics, such as beta-lactam antibiotics, significantly increases the resistance of MRSA, which poses significant challenges to tackle the problem. Therefore, it is very necessary to develop natural, new, and effective antibacterial substances to replace antibiotics and solve the complex problem of MRSA infection. Naturally active compounds with excellent antibacterial activity, especially those derived from plants, have been given great hope to act as alternatives to antibiotics because they are green, safe, and have no antibiotic resistance. For instance, chlorogenic acid (Lu et al., 2020), berberine (Kong et al., 2010), baicalein (Chen et al., 2021), and others have been shown to exert outstanding antibacterial activity against common pathogenic bacteria. Among these, PA is one of the most important compounds. PA, as a vital phenolic acid, is usually extracted from the roots of *Salvia miltiorrhiza* and is also rich in *Stenolomachusanum (L.) Ching* and *Ilex chinensis Sims* (Zhong et al., 2020). Many studies have shown that PA possesses numerous pharmacological properties, including antioxidant, anti-inflammatory, anti-tumor, anti-atherosclerosis and antibacterial activities (Zhong et al., 2016). Up to now, PA has been shown to have a relatively broad antibacterial spectrum, such as *Yersinia enterocolitica* (Meng et al., 2022), *Listeria monocytogenes* (Liao et al., 2023), and *Ralstonia solanacearum* (Li S. et al., 2016), but there is almost no study on the antagonistic activity of PA against MRSA.

In addition, the combination of antibiotics and plant ingredients or native antibacterial compounds provides a feasible strategy to enhance the antibacterial effects and solve the antibiotic resistance problem. For example, studies have reported that combining antibiotics and plant ingredients can synergistically improve the anti-inflammatory effect and antioxidant capacity (Farhat and Khan, 2022). Yu proposed that compound Qingre granules could be combined with vancomycin to synergistically enhance the inhibitory effect on MRSA and improve the sensitivity of vancomycin to MRSA (Yu et al., 2010).

In our study, firstly, we investigated the antibacterial and antibiofilm activities of PA on MRSA. Secondly, the effects of PA on the resistance of MRSA to ampicillin were explored. Finally, the mechanism of mode was investigated and discussed by a series of experiments. This study will help to promote the application of PA in food preservation and infection prevention and pave the way to solve the antibiotic resistance problems.

## 2 Materials and methods

### 2.1 Bacterial strains and chemicals

MRSA ATCC 43300 was obtained from the Guangdong Microbial Culture Collection Center (GDMCC, Guangdong, China). Before use, bacteria were activated and cultured in LB broth (*Hopebio*, Qingdao, China) for 12 h at 37°C.

Protocatechualdehyde (98%, CAS 139-85-5) was purchased from the Shanghai Aladdin Biochemical Technology Co., Ltd. (Shanghai, China) and dissolved in sterile water. Crystal violet (CAS 548-62-9) was purchased from *Solarbio* (*Solarbio* Science and Technology Co., Ltd., Beijing, China). An L7012 *LIVE/DEAD BacLight Bacterial Viability Kit* was purchased from *Invitrogen* (Carlsbad, CA, USA). Gentamycin sulfate (CAS 1405-41-0) and ampicillin (CAS 69-53-4) were obtained from Shanghai Macklin Biochemical Co., Ltd. (Shanghai, China). A potassium standard solution (223006-3) was purchased from Guo Biao Testing and Certification Co., Ltd. (Beijing, China). The reactive oxygen species (ROS) Assay Kit (R272916) was purchased from Shanghai Aladdin Biochemical Technology Co., Ltd. (Shanghai, China). The adenosine triphosphate (ATP) Assay Kit (A095-1-1) was purchased from the Nanjing Jiancheng Bioengineering Institute (Nanjing, China). All other reagents were of analytical grade.

### 2.2 Antibacterial activity of PA on MRSA

#### 2.2.1 Poured plate method

The inhibition zone measurement of PA against MRSA was conducted using the poured plate method. Serial dilutions of PA solutions of 2, 4, and 8 mg/mL were tested. Sterile water and gentamicin solution were used as negative and positive controls, respectively. The diameters of inhibition zones were measured after 12 h of incubation at 37°C in a constant temperature incubator. Each experiment was performed in triplicate.

#### 2.2.2 Growth curve

The time-kill kinetics of PA against MRSA was determined using a growth curve. Serial dilutions of PA solutions of 1, 2, 4, 6, 8, and 10 mg/mL were tested. Sterile water and gentamicin (final concentration of 100 μg/mL) were used as negative and positive controls, respectively. An automatic microbial growth curve tester (37°C, 800 rpm, 2 h interval, Jieling Instrument Manufacturing Co., Ltd. Tianjin, China) was used to measure the growth curves of MRSA. Each experiment was performed in triplicate.

### 2.3 Antibiofilm activity of PA on MRSA

#### 2.3.1 Effect of PA on MRSA preformed biofilm

The effect of PA on MRSA-preformed biofilms was investigated according to a previous reported method (Shi et al., 2018) with some modifications. Two hundred microliters of bacterial suspensions in fresh LB broth (10^6^ CFU/mL) were pipetted into a 96-well flat-bottomed polystyrene microtiter plate (*Corning*, USA) and incubated stationarily for 24 h at 37°C to ensure the full adhesion of the biofilms to the plate bottom. Then the biofilms were rinsed thrice with PBS and treated with 200 μL water and 1, 2, 4, 6, 8 and 10 mg/mL PA at 37°C for 30 min with slight shaking. The plates were rinsed with sterile water, fixed with 200 μL methanol for 20 min, and stained with 200 μL 0.1% (w/v) crystal violet for 10 min. Finally, 200 μL 95% ethanol was pipetted into each well for dissolution. The amount of biofilm was measured by OD_595_ using a Multiskan FC Microplate Reader (*Thermo Fishe*r Scientific, USA) after 30 min of incubation. The background of the uninoculated medium was subtracted from the background of the sample. Each experiment was performed in triplicate.

#### 2.3.2 Effect of PA on biofilm formation of MRSA

The effect of PA on the biofilm formation efficiency of MRSA was adapted from Maury et al. (2019). In contrast to the above experiment, 100 μL bacterial suspension in PBS was pipetted into each well, followed by adding 100 μL of water and 2, 4, 8, 12, 16 and 20 mg/mL PA, respectively, to obtain a final bacterial concentration of 10^8^ CFU/mL and final concentrations of PA of 1, 2, 4, 6, 8, and 10 mg/mL. After 24 h of incubation at 37°C, crystal violet staining was carried out as mentioned above. Each experiment was performed in triplicate.

### 2.4 Reduction of MRSA resistance to ampicillin

#### 2.4.1 Determination of MICs

The minimum inhibition concentrations (MICs) of PA and ampicillin on MRSA were measured according to the method described by Qiao and Sun (2014). The total volume of each well was 200 μL, and the bacteria and antibacterial compounds were mixed in equal volumes. The final bacterial concentration of bacteria in each well was 5 × 10^3^ CFU/mL, and the final concentrations of PA and ampicillin both ranged from 0.5 to 1,024 μg/mL. Wells treated with sterile water were used as negative control, and wells without bacteria were used to deduct the background. After incubation, the absorbance at 600 nm was measured. MICs were defined as the lowest concentration that inhibited more than 90% of the bacteria compared to the negative control group. Each experiment was performed in triplicate. The formula for calculating the inhibition rate is as follows:


$$\mathrm{Inhibition}\mathrm{rate}\left(\%\right)=1-\left(\frac{O\,D_{t\,r\,e\,a\,t\,m\,e\,n\,t\,g\,r\,o\,u\,p}-O\,D_{b\,l\,a\,n\,k\,g\,r\,o\,u\,p}}{O\,D_{n\,e\,g\,a\,t\,i\,v\,e\,c\,o\,n\,t\,r\,o\,l\,g\,r\,o\,u\,p}-O\,D_{b\,l\,a\,n\,k\,g\,r\,o\,u\,p}}\right)$$

#### 2.4.2 Checkerboard dilution test

The antibacterial effects of the combination of PA and ampicillin were assessed using the checkerboard dilution test (Yu et al., 2005). The total volume in each well was 200 μL, and serial dilutions of two different antibacterial compounds (100 μL, 1:1) were mixed in wells, making the final concentrations of PA from 4 to 256 μg/mL and the final concentrations of ampicillin from 0.015625 to 8 μg/mL. The final bacterial concentration of bacteria in each well was also 5 × 10^3^ CFU/mL. The wells treated with sterile water were the negative control group, and the wells without bacteria were the blank group. Each experiment was performed in triplicate. After incubation and measurement of absorbance, the minimum inhibition concentration (MIC) was calculated, and the fractional inhibitory concentration (FIC) was calculated by the following formula:


$$\text{FIC}=\frac{M\,I\,C_{P\,A\,i\,n\,c\,o\,m\,b\,i\,n\,a\,t\,i\,o\,n}}{M\,I\,C_{P\,A\,a\,l\,o\,n\,e}}+\frac{M\,I\,C_{a\,m\,p\,i\,c\,i\,l\,l\,i\,n\,i\,n\,c\,o\,m\,b\,i\,n\,a\,t\,i\,o\,n}}{M\,I\,C_{a\,m\,p\,i\,c\,i\,l\,l\,i\,n\,a\,l\,o\,n\,e}}$$

The minimum FIC was defined as the fractional inhibitory concentration index (FICI). Synergy was defined as a FICI of ≤ 0.5, and antagonism was defined as a FICI > 2. A FICI of > 0.5 but ≤ 1.0 was defined as additivity, and a FICI of > 1.0 but ≤ 2.0, was defined as indifference.

### 2.5 Mechanism of action

#### 2.5.1 Binding of PA to MRSA

The binding between MRSA and PA was verified using a Quartz Crystal Microbalance (QCM; QE401-F1719, Q-sense, Biolin Scientific, AB, Finland). First, log-phase bacterial PBS suspensions (10^8^ CFU/mL) were dropped onto the surface of the Au chip and allowed to stand for 24 h at 4°C to allow full adhesion. Then, when 4 mg/mL PA flowed through the Au chip, the frequency shifts [including the resonance frequency (*Δf*) and energy dissipation (*ΔD*)] of the electrode were examined by QCM to determine if there was a combination. A chip without bacteria adhesion was used to exclude the nonspecific binding.

#### 2.5.2 Ultrastructure observation

##### 2.5.2.1 SEM

Changes in the microstructure and morphology of MRSA induced by PA were imaged using scanning electron microscopy (SEM). Log-phase MRSA (10^8^ CFU/mL) was incubated with PA (final concentration of 4 mg/mL) for 30 min and 37°C with constant shaking at 150 rpm. Sterile water treatment was used as a control. The samples were treated according to a previously reported method and finally observed using SEM (JSM-7500F, Hitachi, Japan).

##### 2.5.2.2 TEM

The intracellular alternations of MRSA induced by PA were observed using transmission electron microscopy (TEM). The treatment method for MRSA by PA was the same as that used for SEM. The samples were treated according to a previously published method and finally observed using TEM (JEM-1200EX, JEOL Ltd., Tokyo, Japan).

#### 2.5.3 Bacterial membrane permeability

##### 2.5.3.1 Live/dead fluorescent staining

Protocatechualdehyde-induced changes in the cell membrane permeability of MRSA were evaluated using the *LIVE/DEAD BacLight Bacterial Viability Kit* (*Invitrogen*, USA). According to the manufacturer’s instructions, log-phase MRSA PBS suspensions (10^8^ CFU/mL) were incubated with PA (final concentration of 4 mg/mL) at 150 rpm and 37°C for 0.5, 3, 6, and 9 h, respectively. After incubation, bacteria were collected, washed, and diluted to approximately 10^6^ CFU/mL. Then, 1 mL of bacterial solution was stained with 3 μL mixed dye of PI: SYTO-9 (1:1) in the dark for 15 min at 24°C and visualized by using TCSsp5II laser scanning confocal microscope (LSCM) (*Agilent*, USA) at the maximum an excitation/emission wavelength of 490/635 nm for PI and 480/500 nm for SYTO-9.

##### 2.5.3.2 Nucleotide leakage

Nucleotide leakage was detected to reflect the bacterial membrane permeability of MRSA treated by PA (Liang et al., 2020). Log-phase MRSA PBS suspensions (10^8^ CFU/mL) were incubated with PA (final concentration was 4 mg/mL) for 30 min at 150 rpm and 37°C. Sterile water was used as negative control. The mixtures were filtered through a 0.22 μm membrane. The OD_260_ of the filtrate was measured using an Evolution 201 ultraviolet-visible spectrophotometer (*Thermo Fishe*r Scientific, USA). Each experiment was performed in triplicate.

##### 2.5.3.3 Potassium ion (K^+^) leakage

The concentration of K^+^ released from MRSA after PA treatment was determined using an atomic absorption spectrophotometer at 766.5 nm (TAS-990; Beijing, China) (Lou et al., 2011). First, a standard curve was plotted based on the K^+^ standard samples (0.5, 1, 2, 3, 4, and 5 μg/mL). After performing the same experimental processes described above, the absorbance of K^+^ in the supernatant was measured using an atomic absorption spectrophotometer. The corresponding concentration of K^+^ was calculated using a standard curve. Each experiment was performed in triplicate.

##### 2.5.3.4 ATP leakage

Extracellular and intracellular adenosine triphosphate (ATP) concentrations in MRSA were measured using an ATP assay kit (Nanjing Jiancheng Bioengineering Institute, Nanjing, China). After the incubation and centrifugation steps, the ATP concentration in the supernatants was measured according to the manufacturer’s instructions to determine the extracellular concentration of ATP. The bacteria were then rinsed thrice with PBS, resuspended in 100 μL PBS, and maintained at 100°C for 10 min to lyse the bacteria. The ATP concentration in the above mixture was considered the intracellular concentration of ATP. Each experiment was performed in triplicate.

#### 2.5.4 ROS detection

Oxidative stress in MRSA after PA treatment was evaluated with a ROS assay kit. Log-phase MRSA PBS suspensions (10^8^ CFU/mL) were incubated with PA (final concentration was 4 mg/mL) at 150 rpm and 37°C for 30 min. Sterile water was used as negative control. Followed by centrifugation to collect bacteria and dissolving them in PBS, 1 mL of bacteria solution was stained with 500 μL of 10 μM 2′,7′-dichlorofluorescein diacetate (DCFH-DA) for 1 h in the dark. After cleaning, the fluorescence intensity was measured at an excitation/emission wavelength of 488/525 nm using Microplate reader (Tecan Co., Ltd. Austria). Each experiment was performed in triplicate.

#### 2.5.5 Transcriptomic analysis

To further demonstrate the mechanism of action of PA, transcriptomic analysis was performed as follows. Log-phase MRSA PBS suspensions (10^8^ CFU/mL) were incubated with PA (final concentration 4 mg/mL) at 150 rpm and 37°C for 30 min. Sterile water was used as the control. The total RNA of MRSA was extracted using TRIzol^®^ reagent according to the manufacturer’s instructions (Invitrogen, USA), and genomic DNA was removed using DNase I (Takara, Japan). RNA quality was determined using a 2100 bioanalyzer (Agilent Technologies, USA) and quantified using an ND-2000 spectrophotometer (NanoDrop Technologies, USA). The data generated from the Illumina platform was used for bioinformatics analysis. All analyses were performed using the free online Majorbio Cloud Platform^1^ of Shanghai, Majorbio Bio-Pharm Technology Co., Ltd. Each experiment was performed in triplicate.

A Per1 program was used to select clean reads by removing low-quality sequences (Q-value ≤ 20), reads with more than 5% of N bases (unknown bases), and reads containing adaptor sequences. For gene expression analysis, clean reads were mapped to the reference using Bowtie2.^2^ Differentially expressed genes (DEGs) among different samples were detected using DEseq2.^3^ An absolute fold change > 1.5 and a *P*-value < 0.05 were set as the thresholds to select the significant DEGs. Then, Gene Ontology (GO) and Kyoto Encyclopedia of Genes and Genomes (KEGG) analyses were performed to assign the DEGs to different functional groups. Goatools^4^ and KOBAS 2.0 (see text footnote 4) were used to identify statistically enriched GO terms and enriched pathways, respectively, using Fisher’s exact test. RNA-seq data for MRSA were deposited in the NCBI Sequence Read Archive under accession number PRJNA970403.

To validate the gene expression results, eight genes were selected from MRSA isolates and quantified by qRT-PCR. All primers used for real-time PCR analysis are listed in Supplementary Table 1. In addition, data were exported and quantified using the comparative Ct method (2^–ΔΔCt^). Each experiment was performed in triplicate.

### 2.6 Effect of PA on preservation of cooked beef

Methicillin-resistant *Staphylococcus aureus* is a common pathogen in livestock breeding and thus usually brought into meat during the process of processing and transportation (Li L. et al., 2016). Raw beef was firstly sterilized at 121°C for 20 min. Then the beef spiked with MRSA was prepared as follows: pieces of sterilized beef weighed 1 g per piece were soaked in sterile water (control) or 4 mg/mL PA for 1 min, respectively. Then the samples were inoculated with 10 μL of MRSA (1 × 10^4^ CFU/mL), sealed in sterile bags, and stored at 4°C until use. The number of MRSA, and two corruption indicators of volatile basic nitrogen (TVB-N) and thiobarbituric acid reactive substances (TBARS) were measured, respectively, at 0, 3, 6, and 9 days to evaluate the effects of PA on beef preservation. 1 g sample was homogenized with 9 mL PBS (pH 7.0), and 10-fold gradient dilutions were used for measurement of MRSA counts. TVB-N was determined according to Zhang et al. (2011) with minor modifications. 10 g sample was dispersed in 75 mL sterile water and stirred for 30 min, centrifuged at 4,000 *g*/min for 2 min and the supernatant was pipetted to another distilling tube. After that, 1 g MgO was added into the mixture, then immediately measured by Kjeldahl instrument (Kjeltec 8400, Danfoss, Denmark). Distilled water was used as control. The distillate was collected in a flask containing 30 mL boric acid solution (20 g/L) and 0.5 mL of mixed indicator of methyl red and bromocresol green. The mixed indicator was freshly prepared before use by mixing methyl red (1 g/L, dissolved in ethanol) and bromocresol green (1 g/L, dissolved in ethanol) at ratio of 1: 5 (v/v). The boric acid solution was titrated with 0.01 mol/L of hydrochloric acid solution. The TVB-N value was determined according to the consumption of hydrochloric acid. TBARS was determined as described by Yarnpakdee et al. (2012) with minor modifications. 1 g sample was mixed with 10 mL extraction solution containing 7.5% trichloroacetic acid (w/v) and 0.1% (w/v) disodium ethylenediamine tetraacetate, shaken at 50°C for 30 min. After filtration, 5 mL filtrate was mixed with 5 mL 0.288% (w/v) thiobarbituric acid, heated 90°C for 30 min to develop a pink color, cooled at running tap water. Then the absorbance value at 532 nm was measured. The same of sterile water instead of sample was used as the control. A standard curve was plotted using 1, 1, 3, 3-tetra ethoxy propane.

### 2.7 Statistical analysis

All experiments are reperformed in triplicate. Data are represented as mean ± SD. One-way analysis of variance (ANOVA) was performed using SPSS version 26 (SPSS Inc., USA). Statistical significance was set considered at *P* < 0.05.

## 3 Results

### 3.1 Antibacterial activity of PA on MRSA

#### 3.1.1 Poured plate method

**FIGURE 1:**
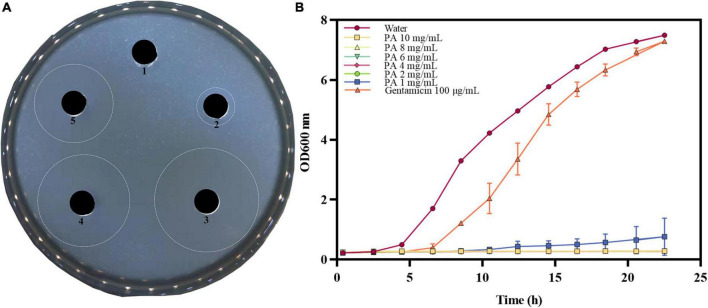
Antibacterial effect of PA against MRSA. **(A)** Inhibition zones by water (1), 100 μg/mL gentamicin (2), 8 mg/mL PA (3), 4 mg/mL PA (4), 2 mg/mL PA (5); **(B)** Growth curves of MRSA treated with water, gentamicin, and different concentrations of PA.

The *in vitro* antibacterial results for different concentrations of PA against MRSA are shown in Figure 1A, and the results of inhibition zone diameters are shown in Supplementary Table 2. As shown in Figure 1A, PA at 2, 4, and 8 mg/mL exhibited prominent inhibition zones against MRSA. In contrast, the water treatment group did not have an inhibition zone, proving that PA could effectively inhibit the growth of MRSA. Moreover, with the increase of the PA concentration, the diameter of the inhibition zone also significantly increased (*P* < 0.05), suggesting that the antibacterial activity of PA against MRSA was dose-dependent manner.

#### 3.1.2 Growth curve

The growth curves of MRSA treated with different PA concentrations are shown in Figure 1B. Compared to the negative control group, the values of OD_600_ significantly dropped (*P* < 0.05) in all PA treatment groups. All the PA treatment groups could completely inhibit MRSA growth, as shown by the horizontal lines in the Figure 1B, except for the 1 mg/mL PA treatment group, which showed a slight growth trend of MRSA after 12 h of culture. In conclusion, the antibacterial effect of PA on MRSA was dose and time dependent, and after comprehensive consideration, 4 mg/mL PA was selected as the optimal treatment concentration for subsequent experiments.

### 3.2 Antibiofilm activity of PA on MRSA

**FIGURE 2:**
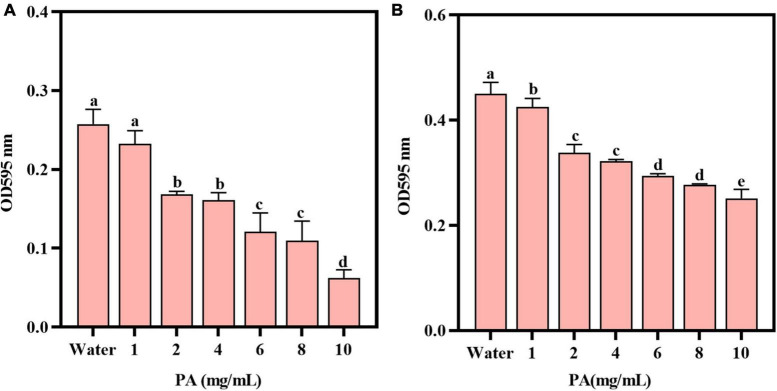
Effects of PA on MRSA biofilms. **(A)** Effect of PA on the established biofilms; **(B)** Effect of PA on MRSA biofilm formation. a, b, and c mean that the data is different and significantly different from each other.

The toxicity and drug resistance of bacteria are closely related to the formation of biofilms (Chen et al., 2022). The antibiofilm activity of PA on MRSA was verified by two experiments. Compared with the control group, PA could significantly reduce (*P* < 0.05) the biomass of the established biofilm of MRSA in a dose-dependent manner (Figure 2A), and the inhibition rate of 4 mg/mL PA was 37.4%. Consistently, compared with the control group, PA (except 1 mg/mL PA) significantly inhibited (*P* < 0.05) the formation of MRSA biofilms in a dose-dependent manner (Figure 2B), and the inhibition rate of 4 mg/mL PA was 28.5%.

### 3.3 Reduction of the resistance of MRSA to ampicillin

When used alone, the MICs of PA and ampicillin against MRSA were 256 μg/mL and 4 μg/mL, respectively. However, in the checkerboard dilution test, when used jointly, one of the two compounds could significantly reduce the MIC value of the other one (Table 1). Specifically, the MIC of PA against MRSA was decreased to 64 μg/mL, and that of ampicillin was decreased to 1 μg/mL. Furthermore, the combination of the two compounds resulted in a lower FICI of 0.5, indicating that there was a synergistic antibacterial effect between PA and ampicillin. These results suggest that PA can significantly reduce the resistance of MRSA to β-lactam antibiotics like ampicillin.

**TABLE 1:** Synergistic antibacterial effect of PA with ampicillin against MRSA.

Name of strain	Compounds name	MIC (μg/mL)		FICI	Combined action
		Alone use	Joint use		
MRSA	PA	256	64	0.5	Synergistic bacteriostasis
	Ampicillin	4	1		

FICI ≤ 0.5 is synergism, FICI > 4 is antagonism, 0.5 < FICI ≤ 4 is an irrelevance.

### 3.4 Mechanism of action

#### 3.4.1 Binding of PA to MRSA

**FIGURE 3:**
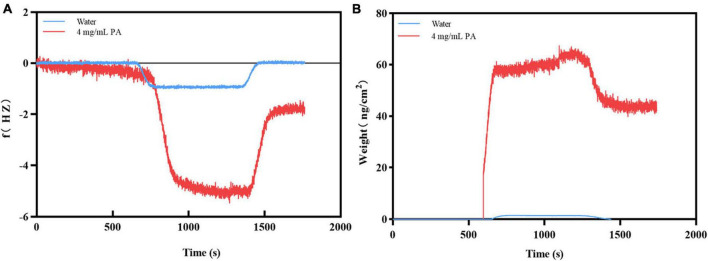
Verification of the binding of PA to MRSA. **(A)** The frequency changes during binding; **(B)** The weight changes during binding.

When PA solution flowed through the surface of the Au chip with MRSA, the frequency increased dramatically until a stable signal strength was reached. After adding ultrapure water for flushing, the frequency decreased sharply, the signal strength reached another stable plateau (*f* value of -1.7962 Hz) (Figure 3A). The trend of the weight signal on the Au chip was the same as that of *f* (weight was 45.90 ng/cm^2^) (Figure 3B). In contrast, when the PA solution flowed through the Au chip, the frequency of the chip increased sharply until a stable signal intensity was reached, but the frequency dropped sharply to a zero baseline when rinsed with ultrapure water (Figure 3A), indicating that the PA solution had no interaction with the Au chip. However, the negative control’s weight signal was almost unchanged throughout the process (Figure 3B). These results suggest that changes in *f* and weight may be the result of the interaction between PA and MRSA, rather than non-specific binding.

#### 3.4.2 Ultrastructure alternations of MRSA after PA treatment

##### 3.4.2.1 SEM

**FIGURE 4:**
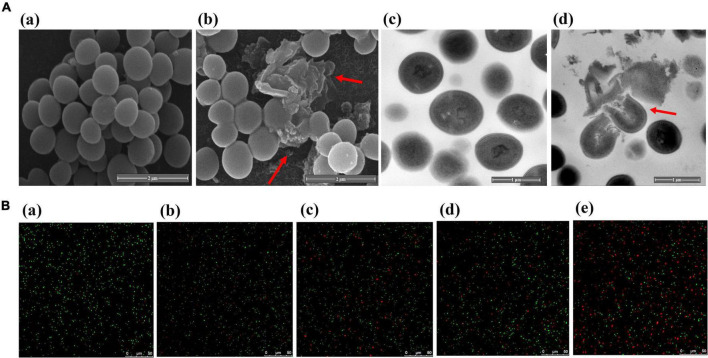
Changes of bacterial integrity and permeability after PA treatment. **(A)** SEM (a, b) and TEM (c, d) images of MRSA before and after treatment with PA; **(B)** LSCM images of MRSA after PA treatment for different times.

Scanning electron microscope (SEM) was used to reveal microscopic changes in the external structure of MRSA after PA treatment (Figure 4A). In the control group, untreated MRSA was spherical or ellipsoidal with smooth surfaces. In contrast, MRSA after PA treatment showed an irregular shape, surface depressions and folds, and a rough surface. Additionally, the bacteria ruptured and aggregated, thereby damaging their integrity.

##### 3.4.2.2 TEM

Transmission electron microscope (TEM) was used to examine the internal structural alterations in MRSA (Figure 4A). The untreated MRSA was spherical and complete. However, after PA treatment, the bacteria were lysed, their entire structure was destroyed, and their cytoplasm leaked.

#### 3.4.3 Bacterial membrane permeability of MRSA affected by PA

##### 3.4.3.1 PI/SYTO-9 staining

PI and SYTO-9 are two nucleic acid dyes. When PI is used alone, it can only penetrate bacteria with ruptured cell membranes, bind nucleic acid and exhibit red fluorescence. In contrast, when SYTO-9 is used alone, it can penetrate both intact and damaged bacteria and emit green fluorescence. However, when combined, the red fluorescence from PI in damaged cells can conceal the green fluorescence from SYTO-9. In summary, more bacteria with red fluorescence reflect higher membrane permeability.

The effects of PA on the bacterial membrane permeability of MRSA are shown in Figure 4B. In the control group, almost all bacteria showed green fluorescence. In contrast, a few bacteria exhibited red fluorescence after PA treatment, indicating that PA treatment could induce an increase in the membrane permeability of MRSA. Meanwhile, with the extension of the PA treatment time, we observed more red bacteria, which suggested that the effects of PA on the bacterial membrane permeability of MRSA were time dependent.

##### 3.4.3.2 Leakage of intracellular components

**FIGURE 5:**
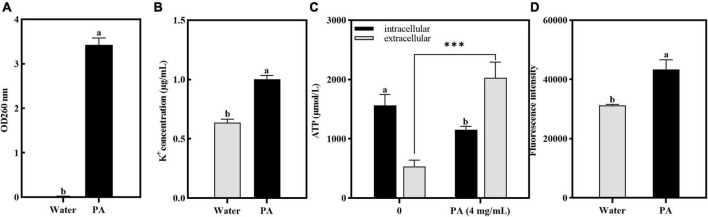
Effects of PA on intracellular components leakage and ROS contents in MRSA. **(A)** Effect of PA on nucleotide leakage; **(B)** Effect of PA on K^+^ leakage; **(C)** Effect of PA on ATP leakage; **(D)** Effect of PA on intracellular ROS contents. a, b, and c mean that the data is different and significantly different from each other. ***Represents a significance level between sample data of 0.001.

As shown in Figure 5A, after treatment with PA, the OD_260_ of the cell-free supernatant increased significantly (*P* < 0.05), indicating that the PA treatment induced the increase of membrane permeability and resulted in the DNA leakage.

Atomic absorption spectrophotometry was used to verify the effect of PA on potassium ion leakage, and the results are shown in Figure 5B. Compared with the control group, the concentration of K^+^ leaked from MRSA was significantly increased (*P* < 0.05). This showed that PA changed the permeability of the cell membranes and promoted the release of K^+^ from MRSA cells.

The effect of PA on the intracellular and extracellular ATP contents of MRSA is shown in Figure 5C. Compared to the control group, the intracellular ATP contents of MRSA treated with PA significantly decreased (*P* < 0.05), whereas the extracellular ATP contents significantly increased (*P* < 0.05), indicating that PA caused MRSA damage and ATP leakage.

#### 3.4.4 ROS detection

In this study, to determine the amount of ROS produced by MRSA after PA treatment, the resulting intracellular ROS was determined using the DCFH-DA method. Figure 5D showed that after PA treatment, the intracellular ROS increased significantly (*P* < 0.05), indicating that the antioxidant defense system of MRSA was imbalanced and had entered a state of oxidative stress.

#### 3.4.5 Transcriptomic analysis of MRSA after PA treatment

**FIGURE 6:**
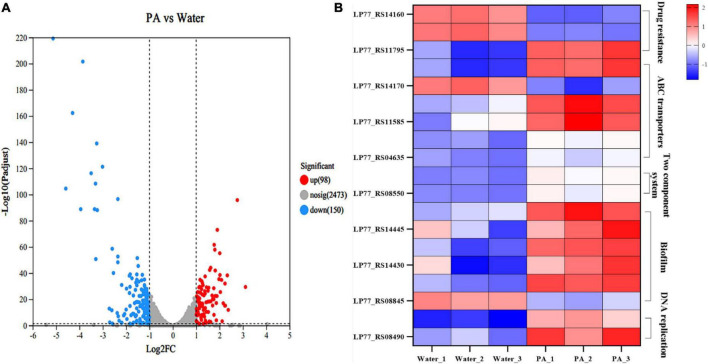
Transcriptome analysis of MRSA after PA treatment. **(A)** Volcano plot of genes expression in MRSA after PA treatment. **(B)** DEGs in different pathways in MRSA after PA treatment, including drug resistance, ABC transporters, two-component system, biofilm formation, and DNA replications impair associated pathways.

Strand-specific prokaryotic transcriptome sequencing was performed to further investigate the antibacterial mechanism of PA against MRSA. In total, 151 million clean reads were obtained, and over 97.14% of the clean reads were mapped to the reference genome sequences (Supplementary Table 3). Correlation and principal component analyses (PCA) revealed good biological duplications and clear cluster between the control and treatment groups (Supplementary Figure 1). Counts of expression genes (FPKM > 1) identified 2,721 genes expressed in MRSA. Among these genes, 248 DEGs in PA-treated MRSA were founded compared to the control group, with 98 genes significantly upregulated and 150 significantly downregulated (Figure 6A; Supplementary Figure 2).

GO, and KEGG enrichment analyses were performed based on these DEGs to obtain a comprehensive explanation (Supplementary Figures 3, 4). A total of 127 GO terms, including 38 (29.92%) molecular function terms, 89 (70.08%) biological process terms, were assigned to the 248 DEGs in MRSA. The top 20 enriched GO terms are shown in Supplementary Figure 3. Among the 20 enriched GO terms, seventeen were related to biological processes, and three to molecular function. In the KEGG enrichment analysis, DEGs were allocated to 88 pathways. The top 20 enriched KEGG pathways are shown in Supplementary Figure 4. The two-component system pathways and Purine metabolism pathways showed the most significant differences among the top 20 enriched pathways.

In the KEGG enrichment analysis, a group of important genes among the 248 DEGs appeared in several important pathways, such as drug resistance (2 DEGs), ATP-binding cassette (ABC) transport systems (5 DEGs), two-component system (3 DEGs), biofilm-related (5 DEGs), and DNA replication repair pathways (2 DEGs), which are closely related to the growth, death and physiological activities of MRSA (Figure 6B).

##### 3.4.5.1 Genes associated with bacterial drug resistance

In the pathway related to MRSA resistance, several significant DEGs were involved. Specifically, drugs such as β-lactam can enter bacteria through the porin encoded by *ompF*, inhibit bacterial growth (Majewski et al., 2016). In this experiment, *ompF* (ILP77_RS14160) was significantly upregulated, indicating that the porin increased or the cavity of the porin enlarged, which might result in the increase of bacterial membrane permeability, thus allowing more antibiotics to enter the bacteria, and reducing the resistance of MRSA to antibiotic. *MprF* is a polypeptide resistance factor and encodes a polypeptide transferase, which is the main cause of resistance of MRSA to vancomycin and daptomycin (Thitiananpakorn et al., 2020). It can catalyze the exchange reaction of L-lysine residues on phospholipid molecules, thus transferring positively charged lysine residues to the carboxyl group of phospholipid to form positively charged lysylphosphatidylglycerol (LysPG) that is a major component of cell membranes and can both affects the stability of cell membranes and increases the resistance of bacteria to cationic antimicrobial peptides (Andra et al., 2011). Herein, the PA treatment resulted in a significant downregulation of *mprF* (ILP77_RS11795), which might lead to the decrease of LysPG synthesis, and then the downregulation of LysPG might affect the stability of cell membrane negatively. To sum it up, PA could decrease the antibiotic resistance of MRSA by regulating a series of genes expression related to antibiotic resistance, which was consistent with the results of the resistance assays to ampicillin above.

##### 3.4.5.2 Genes associated with ABC transport system pathways

The ABC transport system is essential for bacteria to transport nutrients and discharge waste and related to many metabolism pathways such as bacterial drug resistance, membrane permeability, virulence factors, and bacterial growth and death. OPP (Oligopeptide Permease) system is an important part of ABC transport system, which can affect various important physiological processes of bacteria through transport of signal peptides. Both *pstA* and *pstC* encode components of the phosphoric acid transport system, which is a plasma membrane protein complex widely presents in bacteria and involved in the absorption and utilization of exogenous phosphates. PstA, together with PstB, PstC and other proteins, forms a transmembrane protein complex and participates in the active transport of phosphate. PstB is involved in the proton pump role in the energy supply and transport process (Rao and Torriani, 1990). The combined action of PstA and PstC enables bacteria to efficiently absorb and utilize exogenous phosphates, maintaining bacterial growth and metabolism. After PA treatment, *pstA* (ILP77_RS11590), *pstB* (ILP77_RS11595) and *pstC* (ILP77_RS11585) were all significantly downregulated, indicating the significant decrease of transmembrane proteins expression, and then leading to the collapse of the phosphoric acid transport system. *BecA* and *bceB* encode ABC transporters, which can help bacteria to transfer substances harmful to bacteria, such as bacitracin (Ohki et al., 2003). For example, they can transfer bacitracin directly from lipid bilayers to extracellular environment (Bolhuis et al., 1996). In this experiment, both *becA* (ILP77_S04640) and *becB* (ILP77_S04635) were significantly downregulated, which might reduce the ability of transporters to transport antibiotics and harmful substances out of bacteria, and thus have a greater impact on bacterial resistance.

##### 3.4.5.3 Genes associated with two-component system pathways

As a sensing pathway, the two-component system is an important link for bacteria to perceive and adapt the external environment to protect themselves. *VraS* and *vraR* are a pair of two-component regulatory factors in bacteria and related to bacterial drug resistance. This regulatory system is present in many Gram-positive bacteria, such as *S. aureus* (Boyle-Vavra et al., 2006). Deletion of *vraS* or *vraR* has been shown to resensitize *S. aureus* to beta-lactam and vancomycin (Tajbakhsh and Golemi-Kotra, 2019). VraS is a kind of histidine kinase, which can activate VraR by transducing signals into cells when it is sensed by stress factors such as antibiotics. Then the activated VraR binds to the promoter of the target gene *mprF*, thereby regulating the gene transcription level (Tajbakhsh and Golemi-Kotra, 2019). The regulation of *mprF* by VraS/VraR systems is one of the important mechanisms of bacterial resistance regulation (Tajbakhsh and Golemi-Kotra, 2019), which can regulate the transcription level of a variety of genes related to bacterial resistance, thus affecting the sensitivity of bacteria to antibiotics. In this study, both *vraS* (ILP77_S08545) and *vraR* (ILP7_S08550) were significantly downregulated, meaning the regulatory system was disrupted, and the drug resistance of MRSA was weakened, which was consistent with the results of the resistance assays to ampicillin above.

##### 3.4.5.4 Genes associated with bacterial biofilm-associated pathways

Five genes were differentially expressed in the pathway of biofilm formation. Bacterial biofilms are composed of exopolysaccharides and lipoteichoic acids (LTAs). For LTAs formation, there are four essential genes, namely, *dltA*, *dltB*, *dltC*, and *dltD.* It has been proved that deletion of the *dltA* could directly affect the formation of LTAs and significantly reduce the formation of bacterial biofilms (Liu and Hou, 2018). Moreover, *dltA* encodes a part of the D-alanylation system that is in charge of adding a D-alanyl group to the bacterial cell wall, a process called D-alanylation, which can increase the stability of the bacterial cell wall, thereby increasing bacterial resistance to antibiotic (Coupri et al., 2021). Herein, the four genes of *dltA* (ILP77_RS14440), *dltB* (ILP77_RS14435), *dltC* (ILP77_RS14430), and *dltD* (ILP77_RS14425) were all significantly downregulated, which maybe lead to the negative effects of decreasing cell wall stabilization, inhibiting MRSA growth and bacterial biofilm formation. *ArtQ* encodes an arginine ABC transporter permease and is closely related to bacterial colonization and adhesion (Jiang et al., 2021). Some studies have also found that mutation or deletion of *artQ* inhibited the intake of antibiotics, leading to the increased bacterial resistance to antibiotics (Smith et al., 2019). Under the stimulation of PA, *artQ* (ILP77_RS08845) was significantly upregulated. Therefore, it is speculated that the upregulation of *artQ* would promote the intake of antibiotics, resulting in decrease of drug resistance.

##### 3.4.5.5 Genes associated with genetic information processing pathway

DNA polymerase III holoenzyme is a multi-subunit enzyme complex, which contains at least seven different subunits. Of which, α subunit encoded by *dnaE* is the polymerase III core, and ε subunit encoded by *dnaQ* is responsible for correcting the 3′-5′ DNA synthesis activity of the polymerase. After PA treatment, *dnaQ* (ILP77_RS08490) and *dnaE* (ILP77_RS09670) were significantly downregulated, indicating the inhibition of DNA polymerase III synthesis and bacterial DNA replication.

qRT-PCR further suggested that the expression of the selected genes was consistent with the trend of the RNA-seq results (Supplementary Figure 5), indicating that RNA-seq was properly performed and validating the genetic evidence from the transcriptional profiling. The figure was drawn by Figraw.

### 3.5 Effect of PA on beef preservation

**FIGURE 7:**
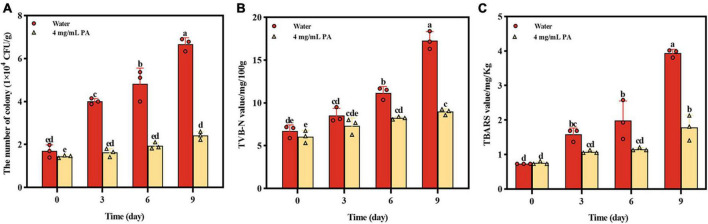
Effects of PA on beef preservation. **(A)** The number of MRSA in cooked beef during storage; **(B)** TVB-N contents in cooked beef during storage; **(C)** TBARS contents in cooked beef during storage. a, b, and c mean that the data is different and significantly different from each other.

The effect of PA on beef preservation was shown on Figure 7. During storage, the growth of MRSA in beef was significantly (*P* < 0.001) inhibited by PA, and the number of MRSA in beef treated by PA was reduced by 63.9% compared with control at day 9 (Figure 7A). TVB-N which is mainly composed of ammonia and primary, secondary, and tertiary amines and TBARS which is produced due to the decomposition of hydroperoxides into secondary oxidation products of lipids are widely used as indicators of meat deterioration. At day 9, the contents of TVB-N and TBARS in PA treated sample were reduced by 48.09% and 54.82%, respectively, compared with the control sample (Figures 7B, C). In general, the results powerfully proved the effective effect of PA on beef preservation, indicating the potential of application in food preservation.

## 4 Discussion

In recent years, the increase in bacterial resistance has broadened research on active antibacterial ingredients. Therefore, they have great potential as substitutes for antibiotics. In this study, the antibacterial and antibiofilm activities of PA against MRSA were investigated, and the synergistic effect of PA with ampicillin against MRSA was evaluated. Meanwhile, the mechanism of antagonistic action was systematically elucidated.

Pouring plate and growth curve methods are the two most used methods for testing the antibacterial activity of certain compounds. In this study, the two methods were all used to test the antibacterial activity and determine the proper concentration of PA used in subsequent assays. The inhibition zone experiment showed a prominent inhibition zone when PA ≥ 2 mg/mL but a stronger inhibition zone when PA ≥ 4 mg/mL. Consistently, in the growth curve experiment, MRSA growth was completely inhibited when PA concentration was ≥ 2 mg/mL. Given this, 4 mg/mL PA was selected as the optimal concentration for subsequent experiments. As we know, many native active compounds from plants have been proved to possess good antibacterial activities, such as chlorogenic acid, gallic acid, ferulic acid, berberine, and other plant extracts. Chen et al. (2022) reported that at least 8 mg/mL of CA could exert inhibition effect on *Y. enterocolitica*. Borges et al. (2012) investigated the antibacterial activities of two phenolic acids, gallic acid and ferulic acid. It has been reported that the minimum bactericidal concentrations for *E. coli* was 2.5 mg/mL (ferulic acid) and 5.0 mg/mL (gallic acid), for *S. aureus* it was 5.0 mg/mL (ferulic acid) and > 5.0 mg/mL (gallic acid), and for *L. monocytogenes* it was > 5.0 mg/mL (both phenolic acids). Kazemian et al. (2015) reported that the biofilm inhibitory concentration of *Chamaemelum nobile* extract against *Pseudomonas aeruginosa* was 6.25–25 mg/mL, and the MIC was 12.5–50 mg/mL. Kong et al. (2010) reported that 50–300 μg/mL of berberine exerted relative good antibacterial activity on *Shigella dysenteriae*. On the whole, although these native compounds exert different antibacterial activities against different species of bacteria, the effective concentrations of these native antibacterial compounds are mostly at mg/mL level. In comparison, PA possesses a little stronger antibacterial activity.

Many studies have shown that MRSA biofilm is an important reason for its resistance to antibiotics (Chung et al., 2022). Therefore, it is a feasible and prospective method to tackle the antibiotics resistance problems by inhibiting bacterial biofilms. Interestingly, some natural active substances have been proved to effectively inhibit the formation of bacterial biofilms, such as alkaloids, terpenoids, flavonoids (Kali, 2015), having a great application potential in treatment of antibiotic resistance problems. As for PA, one of the important phenolic acids, there is an important significance to investigate the effect of PA on MRSA biofilm. Herein, two assays, that is, the effect of PA on MRSA preformed biofilm and the effect of PA on biofilm formation of MRSA, were performed to investigate the problem from two different angles. It was showed that PA significantly decreased the performed biofilm (Figure 2A) and significantly inhibited the formation of biofilm (Figure 2B), suggesting that PA could weaken the antibiotic resistance of MRSA by exhibiting its antibiofilm activity.

Furthermore, studies have shown that many natural active compounds can be combined with antibiotics to exert synergetic antibacterial effect, thus reducing the use of antibiotics (Yu et al., 2010). As we know, MRSA has strong resistance to many antibiotics such as beta-lactam antibiotics. Therefore, there is an urgent need to develop new and effective antibacterial compounds to solve the antibiotics resistance problem of MRSA. In this study, a checkerboard dilution test was performed to test the synergetic antibacterial effect of PA with ampicillin. The lower FICI proved that the combination of PA and ampicillin exhibited a synergistic effect, suggesting that, apart from the possible alternative to antibiotics, PA can significantly reduce the resistance of MRSA to β-lactam antibiotics like ampicillin. The merits of PA may promote the application of PA in the food preservation and pathogen infection.

It can be speculated that the antibacterial effects against MRSA by PA was achieved through the binding of PA to the bacterial cell surface, or the penetrating of PA into the bacteria. These interactions would induce some changes, i.e., frequency and weight changes, which can be detected by QCM. QCM is an excellent sensor and usually used to detect the interaction of two substances based on its supersensitive detection of the oscillation frequency shifts of the electrode and weight changes (Feng et al., 2017). In our study, the obvious changes of frequency and weight changes captured by QCM (Figure 3) proved the interaction of PA and MRSA. However, further studies and some other methods such as molecular docking, molecular biology and multi-omics are needed to determine the way by which the two targets combine, how the interaction functions, and if the interaction affects other cellular metabolisms.

The mode of action of PA on MRSA was elucidated through several assays. Firstly, as for the structure and integrity, the SEM and TEM results illustrated that PA induced a severe damage of the cell walls and cell membranes and ruptured the bacteria integrity (Figure 4). Secondly, as for the membrane permeability, several parameters, including detection of intracellular dye PI (Figure 4), and leakages of DNA, K^+^ and ATP (Figure 5) powerfully verified the increase of the permeability of cell membrane, which was consistent with the results of SEM and TEM. Thirdly, as for ROS generation, a significant increase of intracellular ROS was detected. As we know, under normal conditions, intracellular ROS is the product of normal physiological metabolism, which is in a state of dynamic equilibrium and will not cause damage to the cells. The penetration of exogenous ROS or the collapse of the internal antioxidant defense system can lead to an obvious increase in intracellular ROS, which can result in the damage of proteins, lipids, and DNA (Xia et al., 2006). Therefore, the high generation of intracellular ROS induced by PA maybe one of the factors leading to the death of MRSA. To sum up, PA exerted its antagonistic activity against MRSA through several ways including damaging the structure, increasing the membrane permeability, inducing excess production of intracellular ROS.

**FIGURE 8:**
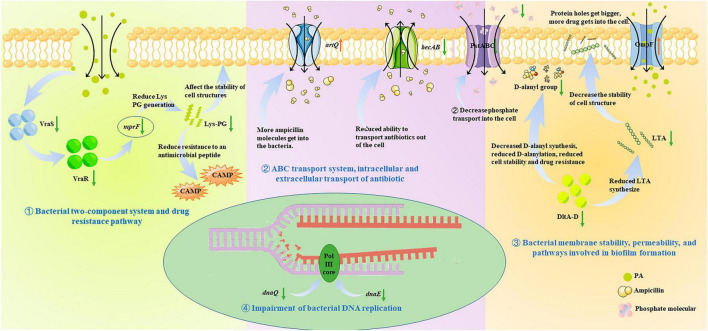
Antibacterial molecular mechanism of PA against MRSA, ① Bacterial two-component system and drug resistance pathway; ② ABC transport system, intracellular and extracellular transport of antibiotic; ③ Bacterial membrane stability, permeability, and pathways involved in biofilm formation; ④ Impairment of bacterial DNA replication.

There is a broad consensus that phenotypic or physiochemical changes are decided or controlled by differential gene expression. Therefore, to further investigate the antibacterial mechanism of PA on MRSA, and to correlate these changes with gene expression, the transcriptome analysis was performed and the roles of the DEGs in some important pathways were analyzed (Figures 6, 8). It was shown that many pathways including two-component system, drug resistance, ABC transport system, biofilm formation, and DNA replication repair associated pathways were negatively affected (Figures 6, 8) because of the abnormal expression of many important genes (Figures 6, 8), leading to the increase of permeability, decrease of drug resistance, damage of biofilms, and impairment of DNA replication. Satisfactorily, the results of transcriptome analysis were consistent with those of phenotypic and physiochemical assays. Combining all the results, it could be concluded that PA exerts antagonistic activity against MRSA through intervening multi-physiological pathways.

Finally, beef spiked with MRSA was employed to test the application of PA in the food preservation. Apart from the significant inhibition of the proliferation of MRSA in beef, the two corruption indicators of TVB-N and TBARS were all significantly reduced compared to the control sample, indicating the application potential in food preservation.

## 5 Conclusion

In our study, it was firstly proved that PA possessed significant antagonistic effects on MRSA, including antibacterial and antibiofilm activities against MRSA. Regarding the mechanism of action, it was demonstrated that PA could destroy the cell membrane and structure integrity, increase the membrane permeability and the intracellular ROS production, and finally leading to the cell death. The lower FICI proved the synergistic effect of PA with ampicillin, suggesting that PA can significantly reduce the resistance of MRSA to β-lactam antibiotics like ampicillin and reduce the use of antibiotics. Moreover, transcriptomics analysis further investigated the mechanism of action and concluded that many pathways including two-component system, drug resistance, ABC transport system, biofilm formation, and DNA replication repair associated pathways were negatively affected. Meanwhile, the inhibition of the growth of MRSA in beef, and the reduction of the two corruption indicators of TVB-N and TBARS showed the application potential in food preservation. Taken together, PA, as an effective antibacterial effector, exerts outstanding antagonistic activity against MRSA and synergy with ampicillin by intervening in many physiological pathways. However, what component of MRSA that PA binds to and how the binding works need to be further studied. In a word, this study will help to promote the application of PA and to solve the problem of MRSA antibiotic resistance.

## Data availability statement

The original contributions presented in the study are publicly available. This data can be found here: https://www.ncbi.nlm.nih.gov/bioproject/; PRJNA970403.

## Author contributions

YW: Conceptualization, Investigation, Methodology, Writing—original draft. XL: Formal analysis, Investigation, Writing—review and editing. LS: Methodology, Resources, Writing—review and editing. KC: Conceptualization, Methodology, Writing—review and editing. CS: Data curation, Methodology, Writing—review and editing. CP: Data curation, Formal analysis, Writing—review and editing. QY: Funding acquisition, Project administration, Writing—review and editing. ZL: Conceptualization, Resources, Supervision, Writing—review and editing.

## Footnotes

1
www.majorbio.com

2
http://bowtie-bio.sourceforge.net/bowtie2/index.shtml

3
http://bioconductor.org/packages/release/bioc/html/DESeq2.htmh

4
https://github.com/tanghaibao/GOatools
