# Supplementary material for: Antibacterial and antibiofilm activities of protocatechualdehyde and its synergy with ampicillin against methicillin-resistant Staphylococcus aureus
Source: Front Microbiol. 2024 Feb 28;15:1366400. doi: 10.3389/fmicb.2024.1366400 (PMC10932990; doi:10.3389/fmicb.2024.1366400)
Supplement: Supplementary file 1 [file Data_Sheet_1.DOCX]

Supplementary Material

**Table S1.** Sequence of primers for MRSA.

| Genes | Forward primer | Reverse primer |
| --- | --- | --- |
| *gyrB* | TTAGTGTGGGAAATTGTCGATAAT | AGTCTTGTGACAATGCGTTTACA |
| *ILP77_RS04735* | CGCAGCCACCTAGATCCTATTACAC | TCCAAACCCTTCGATGCTTCCTTTG |
| *hisD* | AGTGTGGGTATTTATGTGCCTGGTG | TACTCCGTTAGGTTGAGGTGGTGTC |
| *ILP77_RS06730* | GGCGGAGAAATCGGTTCTACTTGG | ACGTGACCAAATGGTCCTTCAGAAG |
| *gltB* | AAGCCTGGTGAAGGTGGTCAATTAC | ATGTGGCGGTGGTGAAATCAGAC |
| *ILP77_RS01950* | AACAACCAGCAGCCGCAGTATTAG | AAGCCTGTAATGTCGTGTCAGTTCC |
| *purS* | ACGCAAGGACAAACGCTTACT | CATCACTAACCTCATCCACTGTCA |
| *grpE* | GCAATAGAAGAAACGTCTGACGAG | TTCAAACTCAGCGTAGAGCCTT |
| *ILP77_RS13750* | GAAAAGCGAAGCGCATTTTCTCAAC | ACCTGCCATTGTGTCTTTCTTAGCC |

**Table S2.** The diameters of the inhibition zone of different concentrations of PA against MRSA.

| Solutions | The diameters of the inhibition zone (mm) |
| --- | --- |
| Water | - |
| 100 μg/mL Gentamicin | 13.4 ± 0.17 |
| 8 mg/mL PA | 31.9 ± 0.294 |
| 4 mg/mL PA | 28.4 ± 0.741 |
| 2 mg/mL PA | 23.1 ± 0.741 |

“-”: No antibacterial effect.

**Table S3.** Summary of RNA-Seq data generated for MRSA samples.

| **Sample name** | **Raw reads** | **Clean reads** | **Clean bases** | **Total mapped (%)** |
| --- | --- | --- | --- | --- |
| Water_1 | 27070482 | 26376556 | 2.64G | 97.39 |
| Water_2 | 23691974 | 23056338 | 2.31G | 97.33 |
| Water_3 | 28550762 | 27596778 | 2.76G | 97.38 |
| PA_1 | 26095698 | 25188958 | 2.52G | 97.31 |
| PA_2 | 26121790 | 25635492 | 2.56G | 97.49 |
| PA_3 | 23876370 | 23104698 | 3.07G | 97.14 |


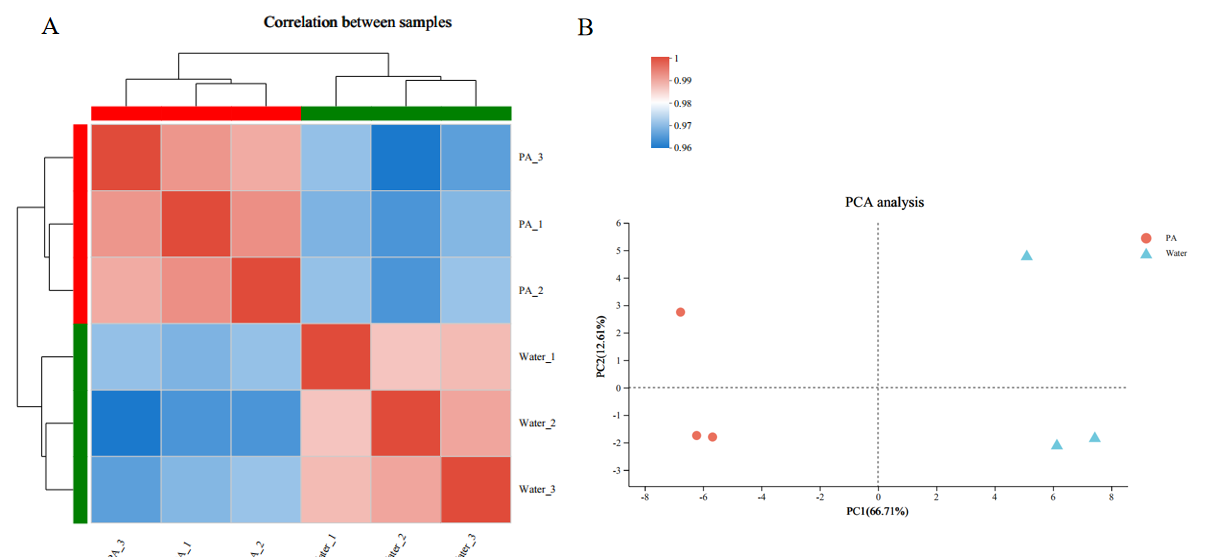


**Supplementary Figure 1.** Sample-to-sample expression analysis. (A) Correlation analysis; (B) Principal component analysis.


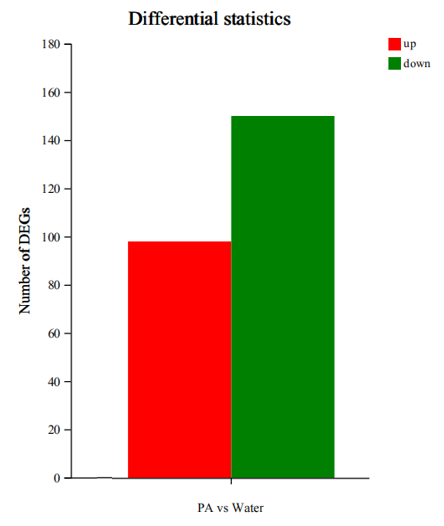


**Supplementary Figure 2.** Differently expressed genes in MRSA after treatment with PA.


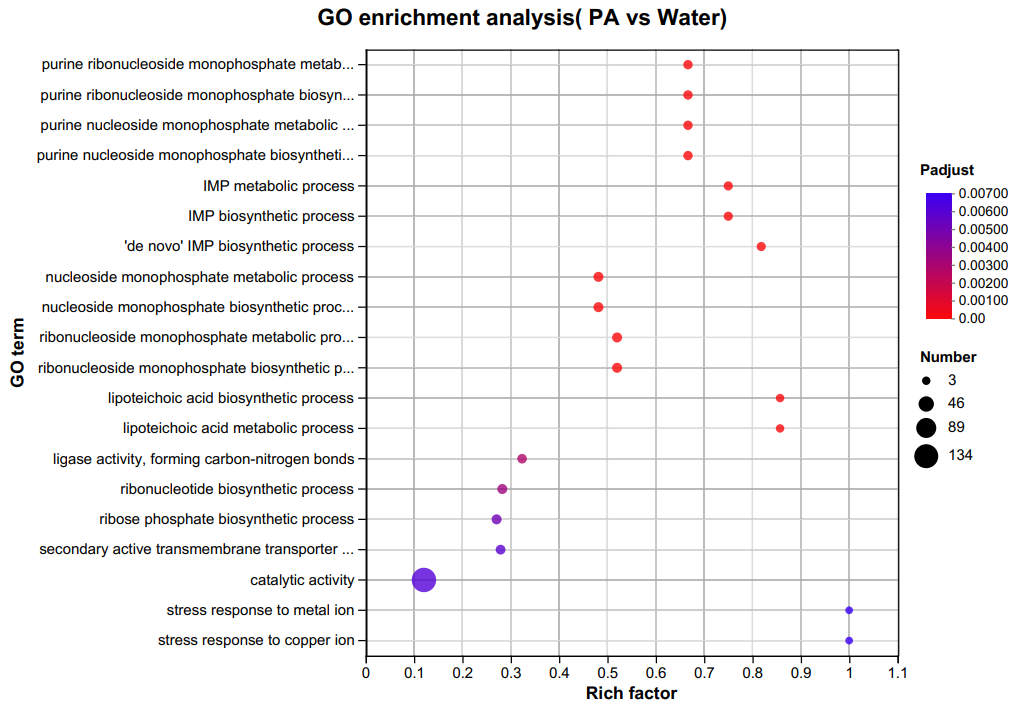


**Supplementary Figure 3.** GO enrichment analysis of DEGs in MRSA after PA treatment.


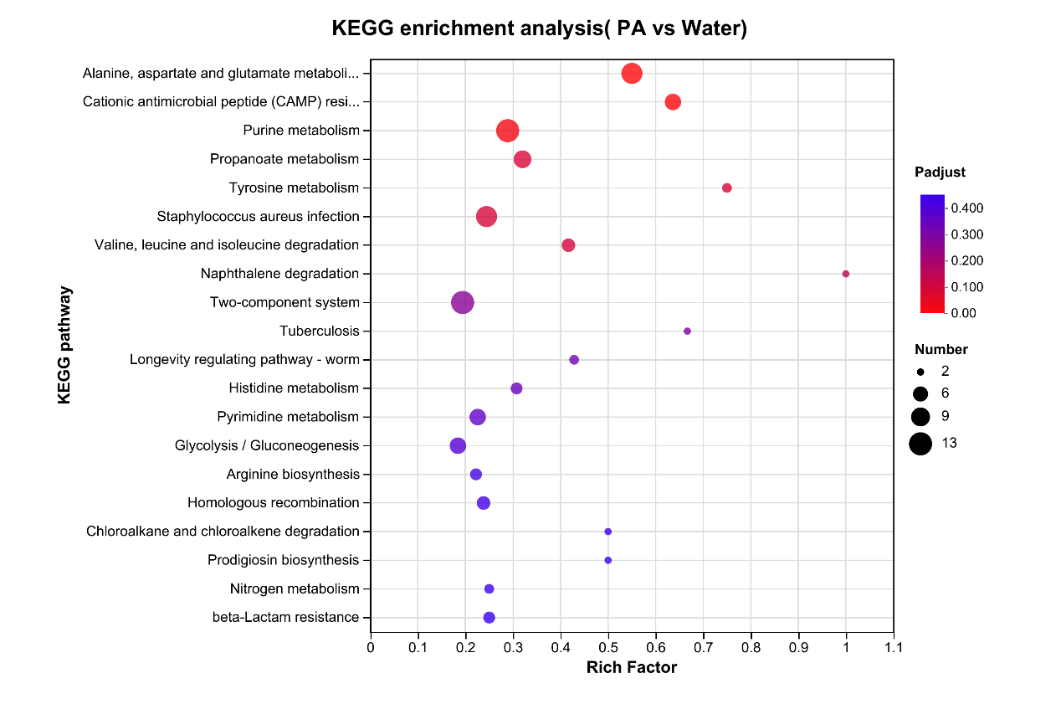


**Supplementary Figure 4.** KEGG enrichment analysis of total DEGs in MRSA after PA treatment.


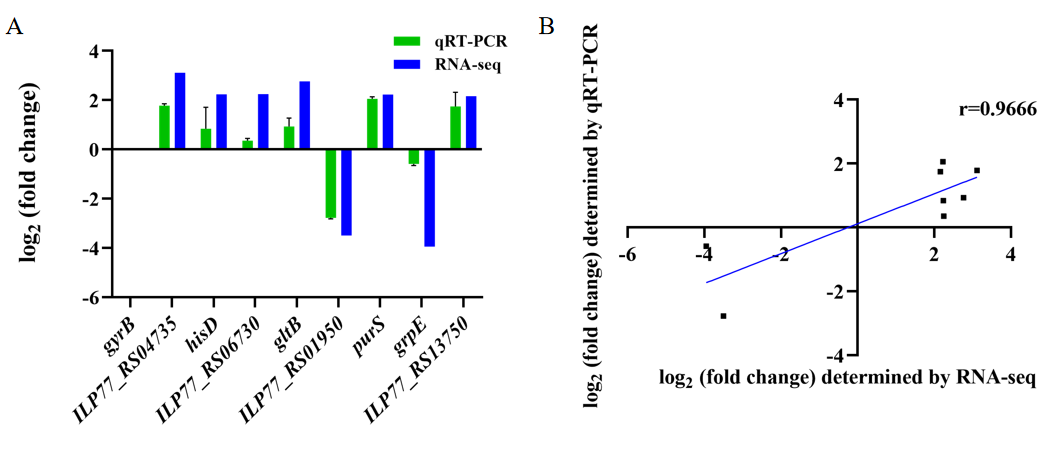


**Supplementary Figure 5.** (A) Q-PCR result; (B) Correlation analysis result.
